# Fortified S-Allyl L-Cysteine: Animal Safety, Effect on Retinal Ischemia, and Role of Wnt in the Underlying Therapeutic Mechanism

**DOI:** 10.1155/2020/3025946

**Published:** 2020-10-03

**Authors:** Windsor Wen-Jin Chao, Yu-Kuang Chen, Howard Wen-Haur Chao, Wynn Hwai-Tzong Pan, Hsiao-Ming Chao

**Affiliations:** ^1^Department of Ophthalmology, Cheng Hsin General Hospital, Taipei, Taiwan; ^2^Institute of Pharmacology, School of Medicine, National Yang-Ming University, Taipei, Taiwan; ^3^Department of Chinese Medicine, School of Chinese Medicine, China Medical University, Taichung, Taiwan

## Abstract

**Purpose:**

Retinal ischemia is a medical condition associated with numerous retinal vascular disorders, such as age-related macular degeneration, glaucoma, and diabetic retinopathy. This in vitro cell and in vivo animal study investigated not only the protective effect of S-allyl L-cysteine (SAC, an active component of garlic) against retinal ischemia but also its associated protective mechanisms.

**Methods:**

Retinal ischemia was mimicked by raising the intraocular pressure to 120 mmHg for 1 hour in one eye. The effects of pre-/postischemic administration of vehicle vs. SAC 0.18 mg vs. SAC 0.018 mg vs. SAC 0.0018 mg treatments on retina cells were evaluated through cellular viability (MTT assay), flash electroretinograms (ERGs), and fluorogold retrograde labelling (retinal ganglion cell (RGC) counting). Also, protein immunoblot was utilized to assess the role of Wnt, hypoxia inducible factor (HIF)-1*α*, and vascular endothelium factor (VEGF) in the proposed anti-ischemic mechanism. Lastly, the safety of drug consumption was investigated for changes in the animal's body weight, ERG waves, and blood biochemical parameters (e.g., glucose levels).

**Results:**

The characteristic ischemic changes including significant reduction in ERG b-wave ratio and RGC number were significantly counteracted by pre- and postischemic low dose of SAC. Additionally, ischemia-induced overexpression of Wnt/HIF-1*α*/VEGF protein was ameliorated significantly by preischemic low dose of SAC. In terms of the animal safety, no significant body weight and electrophysiological differences were observed among defined different concentrations of SAC without following ischemia. In low SAC dosage and vehicle groups, various blood biochemical parameters were normal; however, high and medium concentrations of SAC significantly lowered the levels of uric acid, Hb, and MCHC.

**Conclusion:**

This study shows that preischemic administration of low SAC dosage has been proved to be safe and most effective against rat retinal ischemia electrophysiologically and/or histopathologically. Moreover, counteracting the ischemia-induced overexpression of Wnt/HIF-1*α*/VEGF might presently explain SAC's anti-ischemic mechanism.

## 1. Introduction

Common retinal ischemic disorders include central/branch retinal artery occlusion, central/branch retinal vein occlusion, glaucoma, diabetic retinopathy, and neovascular age-related macular degeneration. These diseases are associated with visual impairment and even blindness in more severe cases. Characteristic retinal ischemic findings are known to affect electroretinogram's b-wave amplitude and retinal ganglion cell (RGC) number [1]. There are also important Wnt pathway-mediated developmental retinal ischemic diseases that include diabetic retinopathy, familial exudative vitreoretinopathy, and persistent hyperplastic primary vitreous [2–6]. However, the Wnt pathway is still an area of active research, which is not completely understood and will be explored throughout this paper.

The current knowledge is that the canonical Wnt pathway is said to stabilize *β*-catenin expression. This is done by binding to frizzled and low-density lipoprotein receptor-related protein 5 or 6, which prevents the breakdown of *β*-catenin [3]. Liu et al. have indicated that *β*-catenin can work with lymphoid enhancer-binding factor 1, in order to regulate the target gene expression as vascular endothelial growth factor (VEGF) [3]. Other studies have shown that the canonical Wnt signaling enhances the expression of hypoxia induced factor-1*α* (HIF-1*α*), which consequently also increases VEGF levels in hypoxia [7–12]. Our previous studies have mentioned that overexpression of Wnt pathway plays a pathogenic role of Norries disease and that blockage of Wnt pathway has been beneficial in its treatment [13].

Therefore, it is important to carry out an investigation into the ischemic alterations associated with the Wnt pathway as well as provide an alternative and complementary treatment to it. Due to the limitations of current treatment (e.g., anti-VEGF therapy, laser photocoagulation, and transpupillary thermotherapy), new strategies for retinal vascular disorders driven by persistent ischemia/hypoxia are needed [14]. In this case, S-allyl L-cysteine (SAC), an active organic compound found in aged garlic extract, has been presently selected as a treatment for research due to its antioxidative, anti-inflammatory, and antiapoptotic properties [15, 16]. Also, the US patent (US8569372 B2) has shown that 3.2∼12.8 × 10^−8^ g/kg≈3∼150 ng of SAC has been proven to protect the human eye against retinal ischemia via its antioxidative and other neuroprotective properties [7, 8]. Lastly, its low toxicity [17] makes it an ideal candidate for the treatment of retinal ischemia-related ocular disorders.

In the following study, the effects and mechanisms of SAC against retinal ischemia-reperfusion (I/R) injury were explored, via cellular viability analysis, electroretinogram measurement along with retrograde fluorogold labelling RGC number count, and also its regulation on the defined proteins of Wnt, HIF-1*α*, or VEGF (identified through western blotting assay). Finally, the safety of fortified SAC in animals was also evaluated through its effects on the animal's body weight (BW), electrophysiology, and biochemical alterations. Overall, it is hypothesized that SAC would be able to nullify ischemic-induced insults in the retina and with little side effects. Specifically, the addition of SAC would downregulate the level of Wnt, HIF-1*α*, and VEGF protein expression. Thus, it would lead to less pronounced retinal ischemia associated alterations, namely, decreased RGC number and ERG b-wave amplitude.

## 2. Materials and Methods

### 2.1. Chemicals and Drug Administration

SAC was purchased from Sigma-Aldrich (MO, USA) and prepared by dissolving it in distilled water. Based on our previous publication [7], the maximal concentration of 0.1 mM of SAC studied is equivalent to an oral dose of 0.18 *μ*g (0.00018 mg) in a 0.2 kg rat and 10 *μ*g of a 70 kg human. This means that the cellular concentration of the fortified (10-fold) SAC (i.e., 1 mM) is equivalent to 0.0018 mg (1.8 *μ*g) per day in animal tests. In the present in vitro cellular viability tests, ten (1 mM), hundred (10 mM), and thousand folds (100 mM) of the original dose were prepared. These are equivalent to 0.0018 (low dose), 0.018 (medium dose), and 0.18 mg (high dose) used in the in vivo animal studies.

For the animal study, defined administration by oral gavage of daily dose of SAC was carried out in these following groups, namely, a preischemic administration (SAC 0.0018 mg + I/R and SAC 0.018 mg + I/R) for 4 weeks or a postischemic administration (I/R + SAC 0.0018 mg) for seven days. In contrast, the rats in the Vehicle + I/R group subjected to ischemia were preadministrated with the same volume of vehicle as that of the pre-/postadministrated SAC.

### 2.2. In Vitro Studies

#### 2.2.1. Oxygen Glucose Deprivation (OGD) and MTT (Viability) Assay

The RGC-5 cell lines for the dosing regimen were prepared using the protocol described by Chao et al. [13] but with minor modifications. In this case, OGD was induced by growing cells in Dulbecco's Modified Eagle's Medium without glucose (DMEM; Sigma-Aldrich) at 37°C, 1% oxygen, 94% nitrogen, and 5% carbon dioxide (set by Penguin Incubator; Astec Company, Kukuoka, Japan). This is a well-known model to mimic retinal ischemia. Then, the cells were divided into different treatment groups, namely, Vehicle + DMEM (control cells), Vehicle + OGD (treated ischemic-like cells), SAC 0.1/0.3/0.5/1 mM + OGD (treated ischemic-like cells in lower SAC concentrations), and SAC 1/10/100 mM + OGD (treated ischemic-like cells in higher SAC concentrations). As defined, 1 mM is equivalent to 0.0018 mg/day utilized in animal tests, and the cells actually had one hour of pre-OGD administration. When the first day of OGD treatment has ended, the RGC-5 cells were placed into new DMEM solutions for another day, and MTT test was carried out.

The MTT assay was carried out, in order to test the efficient concentrations of SAC. When the NAD(P) H-dependent cellular oxidoreductase enzymes reduces MTT, it forms a purple-colored substance called formazan. In this case, a more purple solution reflects a higher number of viable cells. In order to conduct this test, MTT (0.5 mg/mL; Sigma-Aldrich) was placed into 96-well plate that contains 100 *μ*L of experimental cells and allowed to react for a total of 3 h at 37°C. Then, the reduced MTT was solubilized by the addition of 100 *μ*L of dimethyl sulfoxide [7], and the plate was shaken to help with further dissolving. Then, the optical density (OD) of the solution was obtained, via the usage of ELISA reader (Synergy H1 Multimode Reader BioTek Instruments) at 562 nm. Finally, the cell viability was calculated as OD measurements relative to the control, which is set at 100%.

### 2.3. In Vivo Studies

#### 2.3.1. Animals

This animal study followed the regulations, which is set by ARVO Statement for the Use of Animals in Ophthalmology and Vision Research. Also, the permission to conduct the study was obtained from institutional review board of Cheng-Hsin General Hospital (Taipei, Taiwan; Approval No: CHIACUC 106-09). As for the animals, six-week-old Wistar rats (BioLasco) were purchased and reared in an environment set at 40∼60% humidity and 19∼23°C temperature. Throughout the experimental days, the animals were kept on a 12 h light/dark setting along with 12∼15 air refreshment per hour and also given food and water at their own pleasure.

Then, the animals were randomly placed into normal, ischemic (Vehicle + I/R), preischemic treatment groups (SAC 0.0018 mg + I/R and SAC 0.018 mg + I/R) and postischemic treatment group (SAC I/R + 0.0018 mg).

### 2.4. Establishing Retinal Ischemia

#### 2.4.1. *Anesthesia* and *Euthanasia*

An intraperitoneal injection was employed to anesthetize the rats. Specifically, 100 mg/kg ketamine (Pfizer) and 5 mg/kg xylazine (Sigma-Aldrich) are given to the rat, which causes the effect of sedation along with analgesia. Another intraperitoneal injection of 140 mg/kg sodium pentobarbital (SCI Pharmtech) was conducted to humanely kill the rat (Scientific Procedures Acts 1986).

#### 2.4.2. *Ischemia* Induction

Each rat (200–250 g) was anesthetized using the method described above and set onto a stereotaxic frame. As described in Chao et al. [13], a rat model of retinal I/R injury was established through increase of intraocular pressure. Specifically, the cornea of the rat is cannulated using a 30-gauge needle connected to an elevated 0.9% saline reservoir and fixed inside the anterior chamber at one eye for 1 h. By doing so, it creates high intraocular pressure of 120 mmHg and signs of retinal whitening, which is clinically characterized as ischemia. Lastly, the rats were rested upon heating pads, which were set at 37°C throughout ischemia and the subsequent 3 h reperfusion treatment. On the other hand, the control groups were presently nonmanipulated rats.

### 2.5. Blood Collection and Measurement

After the rats were euthanized, blood sample is slowly taken from the rat's left ventricle, via technique known as cardiac puncture [18]. Then, the samples were rested at 22°C for a total of 1 h to clot and then centrifuged at 3000 rpm and 4°C for 10 min. This allows the formation of serum, which is separated and to be tested. The tests included glucose, blood urea nitrogen (BUN), creatinine, aspartate aminotransferase (AST), alanine aminotransferase (ALT), alkaline phosphatase (ALKP), triglyceride, cholesterol, high-density lipoproteins (HDL), low-density lipoproteins (LDL), red blood cell (RBC), hematocrit, platelet, albumin, white blood cells (WBC), uric acid, and mean corpuscular volume/hemoglobin concentration (MCV/MCHC). These levels of these biochemicals were determined by the automatic analyzer (Beckman Coulter AU480, USA).

### 2.6. Flash ERG Measurements

For the flash ERG measurements, they were carried out on day 0 (nonadministered rats) along with the day after vehicle + I/R, SAC + I/R, or I/R + SAC procedure. Then, the dark adaptation was carried out for a total of 8 hours, and anesthesia was conducted for ERG recording with pupil dilation. Then, a stimulus of 0.5 s^−1^ was given via a strobe 2 cm before the rat's eyes. In this case, fifteen continuous recordings were done at each 2 s interval, which is set at 10 s^−1^. Afterwards, their amplitudes were maximized and calibrated to give a mean, via usage of an amplifier P511, regulated power supply RPS107, and stimulator PS22 (Grass-Telefactor). The b-wave amplitude ratio of I/R eye to that of the normal eye was measured, in order to compare between different experimental treatments as illustrated by Chao et al. [13].

### 2.7. RGC Retrograde Staining

The anesthetic rat is placed onto the stereotaxic frame, and a 2 cm deep cut is created on the rat's scalp and 2 small holes drilled into its skull [7]. Afterwards, a micropipette was used to inject 10 *μ*l of 5% fluorogold (Sigma-Aldrich) at 3.8, 4.0, and 4.2 mm below its skull. Of note, this step was carried out 3 days prior euthanasia of rat. Then, the retina was dissected and collected, as described by Chao et al. [7]. Finally, the RGC count was determined through the equation of total RGC number over total area of retina used [7].

### 2.8. Protein Immunoblot

The samples of the retina were isolated and sonicated in lysis buffer (i.e., mammalian protein extraction reagent; Hycell) after the rat is euthanized. Then, they were divided to similar amount of 30 *μ*g/30 *μ*l/well, and underwent separation through 12% sodium dodecyl sulfate polyacrylamide gel electrophoresis (Bio-Rad, Hercules, CA). Once that is completed, the samples were transferred from the gel onto polyvinylidene fluoride membrane. The membranes itself were soaked with 5% fat-free skimmed milk at 4°C and blocking buffer (135 mM NaCl, 8.1 mM Na_2_HPO_4_, 1.5 mM KH_2_PO_4_, and 2.7 mM KCl; pH 7.2) for 16 hours. Then, the membranes were incubated with a series of primary antibodies (Abcam Inc. Cambridge, UK) at 25°C for 1 hour, namely, mouse anti-*β*-actin monoclonal antibody (ab6276; 1 : 5000), rabbit anti-Wnt3a monoclonal antibody (ab172612; 1 : 1000), mouse anti-HIF-1*α* antibody (1 : 200; H1alpha67-ChIP Grade; Abcam Inc.), and rabbit polyclonal anti-VEGF antibody (A-20; 1 : 200; sc-152). The blots were then incubated with the relevant secondary antibodies, which were horseradish peroxidase-conjugated goat anti-rabbit IgG (111-035-003; 1 : 2000; Jackson ImmunoResearch) and anti-mouse IgG (sc-2005; 1 : 2000; Santa Cruz Biotech Inc., California, US) at 25°C for 1 hour. Lastly, the blots were developed through enhanced chemiluminescent analysis system (HyCell) and exposed to an X-ray film (Fujifilm), and then the proteins levels were analyzed through scanning densitometry.

### 2.9. Statistical Analysis

An unpaired *t* test was utilized to compare between two groups, whereas one-way analysis of variance (ANOVA) was carried out for comparison of three or more than 3 independent groups. After the ANOVA tests, Dunnet's test was selected to compare the control (e.g., Vehicle + I/R) versus the rest of the groups (e.g., SAC 0.0018 mg + I/R). Of note, results were presented as mean ± SE, and a *P* value that is smaller than 0.05 was considered as significant.

## 3. Results

### 3.1. Oxygen Glucose Deprivation (OGD) and MTT (Viability) Assay

To test the optimal and safe cellular concentration of fortified SAC onOGD-insulted cells, lower (0.1, 0.3, and 0.5 mM) and higher (1, 10, and 100 mM) concentrations of pre-OGD administration of SAC were tested and then compared against the control group (set as 100%; cells cultivated in culture medium containing vehicle, Vehicle + DMEM).

**Figure 1:**
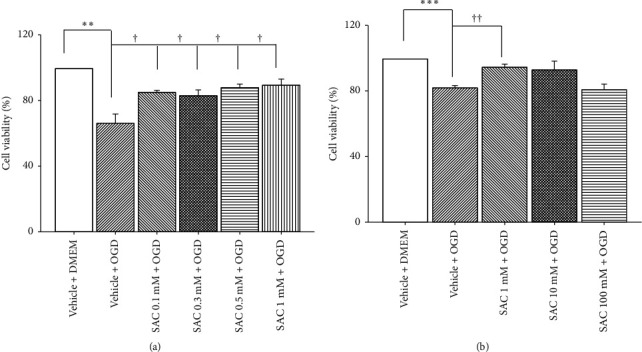
The optimal and safe cellular concentration of fortified SAC on ischemia-induced cell viability as evaluated by MTT assay and a cellular ischemia-like model (i.e., OGD) for 1 day. (a) The viability (%) of cells that were cultivated in culture medium plus one hour of pre-OGD administration of Vehicle or SAC: Vehicle + OGD, SAC 0.1 mM + OGD, SAC 0.3 mM + OGD, SAC 0.5 mM + OGD, and SAC 1 mM + OGD were recorded. As compared with the control group (set as 100%; cells cultivated in culture medium containing vehicle, Vehicle + DMEM), the Vehicle + OGD treatment resulted in significant decrease of cellular viability (^*∗∗*^*P* < 0.05). This decrease was countered by SAC treatment that resulted in a dosage related and significant (^†^*P* < 0.05; at 0.1∼1 mM) attenuation of the OGD-induced cellular injury. (b) The efficiency of SAC on the cells in culture medium plus one hour of pre-OGD administration of Vehicle or SAC: Vehicle + OGD, SAC 1 mM + OGD, SAC 10 mM + OGD, and SAC 100 mM + OGD were measured, in terms of cellular viability (%). As compared with the control group, the Vehicle + OGD treatment resulted in a significant decrease of cellular viability (^*∗∗∗*^*P* < 0.05). However, this decrease was attenuated by SAC treatment, but it only resulted in significant (^††^*P* < 0.05; at 1 mM) attenuation of the OGD induced cellular injury. *Note*. 1 mM is equivalent to 0.0018 mg/day in animal tests. SAC, S-allyl L-cysteine; OGD, oxygen-glucose deprivation.

As demonstrated in Figure 1(a), the Vehicle + OGD had recorded value of 66.56 ± 5.67%, which has the lowest cell viability (%) out of all groups. On the other hand, low concentration SAC treatment resulted in a generally dosage related and significant attenuation of the OGD-induced cellular injury, relative to the Vehicle + OGD group. This corresponds to values of 85.39 ± 1.22% for SAC 0.1 mM + OGD, 83.43 ± 3.52% for SAC 0.3 mM + OGD, 88.42 ± 2.08% for SAC 0.5 mM + OGD, and 89.83 ± 3.74% for SAC 1 mM + OGD.

In contrast, as shown in Figure 1(b), there was not a dose-response relationship from 1∼100 mM. The cell viability generated for groups are as follows: 82.27 ± 1.48% for Vehicle + OGD, 94.89 ± 1.97% for SAC 1 mM + OGD, 93.26 ± 5.38% for SAC 10 mM + OGD, and 81.25 ± 3.38% for SAC 100 mM + OGD. As compared with the Vehicle + OGD group, only 1 mM of SAC treatment resulted in significant (*P* < 0.001; at 1 mM) attenuation of the OGD-induced cellular injury.

Overall, the result implies that SAC with concentrations below 1 mM or 0.0018 mg/day should have some form of protective effect against ischemic injury. Thus, this data along with ERG's results (mentioned later) lead to the selection of SAC 0.0018 mg for testing in animal studies, namely, fluorogold labelling and western blot analysis.

### 3.2. Body Weight (BW) Changes after Oral Gavage for 4 Weeks of Daily Preischemia (Tested before Subsequent Retinal Ischemia) Administration of High/Medium/Low Dose of SAC or Same Volume of Vehicle

As shown in Table 1, there was no significant difference of BW between preischemic daily oral gavage of vehicle and that of 0.18/0.018/0.0018 mg of SAC at each time point, respectively, during an observation period of week 0 (baseline) to week 4. For easier comparison, the weekly data were represented as “vehicle vs. SAC 0.18 mg vs. SAC 0.018 mg vs. **SAC 0.0018 mg**”, which is as follows: baseline (220.2 ± 18.1 vs. 217.0 ± 18.2 vs. 221.8 ± 16.9 vs. **224.0** ± **18.1**), week 1 (258.2 ± 16.0 vs. 257.3 ± 14.5 vs. 262.2 ± 15.8 vs. **269.5** ± **18.2**), week 2 (282.2 ± 13.8 vs. 280.0 ± 8.3 vs. 304.5 ± 14.7 vs. **292.0** ± **12.1**), week 3 (324.0 ± 12.3 vs. 310.7 ± 6.7 vs. 325.7 ± 9.1 vs. **328.2** ± **8.2**), and week 4 (321.0 ± 9.5 vs. 322.3 ± 8.7 vs. 323.3 ± 12.5 vs. **330.7** ± **17.7**). Of note, these BW results were represented as mean ± SE (g). From the values above, it could be implied that the animals followed a normal growth rate over time (increase of BW), which is not affected by the presence of vehicle or SAC.

### 3.3. ERG a/b-Wave Amplitudes after Oral Gavage for 4 Weeks of Daily Preischemia (Tested before Subsequent Retinal Ischemia) Administration of High/Medium/Low Dose of SAC Are Given or Same Volume of Vehicle

By means of ERG, preischemia daily doses of vehicle or SAC (0.18, 0.018, or 0.0018 mg) have been proved to be safe, as shown in Tables 2 and 3. Once again, the data will be represented as “vehicle vs. SAC 0.18 mg vs. SAC 0.018 mg vs. **SAC 0.0018 mg**” across weeks, which enables a comparison between different groups. The ERG a-wave amplitudes in Table 2 were presented as mean ± SE (mV) and listed as follows: for baseline (0.08 ± 0.02 vs. 0.06 ± 0.02 vs. 0.09 ± 0.02 vs. **0.07** ± **0.02**), week 1 (0.10 ± 0.03 vs. 0.09 ± 0.02 vs. 0.07 ± 0.02 vs. **0.05** ± **0.01**), week 2 (0.09 ± 0.03 vs. 0.08 ± 0.02 vs. 0.10 ± 0.02 vs. **0.06** ± **0.02**), week 3 (0.07 ± 0.02 vs. 0.07 ± 0.02 vs. 0.06 ± 0.02 vs. **0.06** ± **0.02**), and week 4 (0.09 ± 0.03 vs. 0.07 ± 0.02 vs. 0.09 ± 0.02 vs. **0.06** ± **0.01**). Throughout the 4 weeks of observation, there was no significant difference of a-wave amplitudes between each group and at each week.

As for Table 3, the ERG b-wave amplitudes were also recorded as mean ± SE (mV) and have values as follows: baseline (0.43 ± 0.03 vs. 0.41 ± 0.05 vs. 0.48 ± 0.06 vs. **0.41** ± **0.07**), week 1 (0.49 ± 0.05 vs. 0.44 ± 0.05 vs. 0.44 ± 0.06 vs. **0.45** ± **0.06**), week 2 (0.43 ± 0.07 vs. 0.37 ± 0.06 vs. 0.46 ± 0.01 vs. **0.47** ± **0.08**), week 3 (0.41 ± 0.07 vs. 0.35 ± 0.04 vs. 0.44 ± 0.06 vs. **0.40** ± **0.01**), and week 4 (0.35 ± 0.04 vs. 0.38 ± 0.05 vs. 0.44 ± 0.03 vs. **0.41** ± **0.04**). Similarly, these data are also not significantly different across different groups and various weeks.

Of note, the ERG a-wave amplitudes are relatively smaller and sometimes harder to be measured than b-wave amplitudes. Thus, it is usually disregarded for the test of therapeutic properties of drugs; instead, it is presently for safety tests and evidence of retinal normality.

### 3.4. Blood Biochemical Measurements after Oral Gavage for 4 Weeks of Daily Preischemia (Tested before Subsequent Retinal Ischemia) Administration of High/Medium/Low Dose of SAC Are Given

As reported in previous publication https://pubmed.ncbi.nlm.nih.gov/31651183/ [19], blood laboratory examinations were carried out to test the drug safety and side effects, via comparing pretreatment of normal laboratory data of blood biochemical parameters to that of posttreatment ones. In this case, the data before and after oral gavages of preischemic vehicle (pre-I/R vehicle or nontreatment) or 0.18/0.018/0.0018 mg of SAC (pre-I/R SAC or SAC treatment) were obtained and expressed as “vehicle vs. SAC 0.18 mg vs. SAC 0.018 mg vs. **SAC 0.0018 mg**” for comparison.

After 4 weeks of treatment, there were no significant differences among various groups (*n* = 4∼11): for glucose (136.55 ± 4.67 vs. 124.71 ± 6.83 vs. 128.76 ± 3.73 vs. 130.39 ± 8.41), blood urea nitrogen (15.89 ± 0.51 vs. 15.39 ± 0.42 vs. 15.75 ± 0.65 vs.17.14 ± 1.25), creatinine (0.41 ± 0.01 vs. 0.43 ± 0.01 vs. 0.42 ± 0.02 vs. 0.47 ± 0.02), AST (109.71 ± 5.91 vs. 104.25 ± 5.46 vs. 112.86 ± 9.12 vs. 107.71 ± 2.30), ALT (35.67 ± 1.62 vs. 31.43 ± 0.84 vs. 33.71 ± 2.16 vs. 34.86 ± 1.72), ALKP (101.40 ± 5.22 vs. 113.00 ± 4.99 vs. 113.17 ± 5.38 vs. 108.14 ± 3.94), triglyceride (39.11 ± 6.08 vs. 35.57 ± 3.47 vs. 38.00 ± 7.48 vs. 35.22 ± 3.78), cholesterol (41.89 ± 2.34 vs. 38.11 ± 3.01 vs. 36.00 ± 2.16 vs. 40.33 ± 1.35), high-density lipoprotein (HDL; 26.71 ± 0.99 vs. 26.13 ± 1.17 vs. 25.00 ± 0.63 vs. 25.67 ± 0.49), low-density lipoprotein (LDL; 7.44 ± 0.29 vs. 9.29 ± 0.57 vs. 7.67 ± 0.67 vs. 10.57 ± 0.53), red blood cells (8.12 ± 0.16 vs. 8.00 ± 0.11 vs. 7.79 ± 0.18 vs. 8.19 ± 0.13), hematocrit (46.03 ± 0.65 vs. 44.04 ± 0.49 vs. 44.94 ± 0.77 vs. 44.24 ± 0.60), mean corpuscular volume (MCV: 55.68 ± 0.72 vs. 55.46 ± 0.50 vs. 55.46 ± 0.85 vs. 54.51 ± 0.67), mean corpuscular hemoglobin (MCH; 19.10 ± 0.27 vs. 18.98 ± 0.15 vs. 19.16 ± 0.31 vs. 18.79 ± 0.18), and platelet (1018.89 ± 33.50 vs. 1022.00 ± 36.15 vs. 1148.50 ± 46.56 vs. 1032.50 ± 69.83). In addition, there were also no significant differences among various groups (*n* = 6∼8) for albumin (1.51 ± 0.04 vs. 1.68 ± 0.04 vs. 1.58 ± 0.03) and WBC (6.19 ± 0.29 vs. 7.140.39 vs. 6.80 ± 0.51).

**Figure 2:**
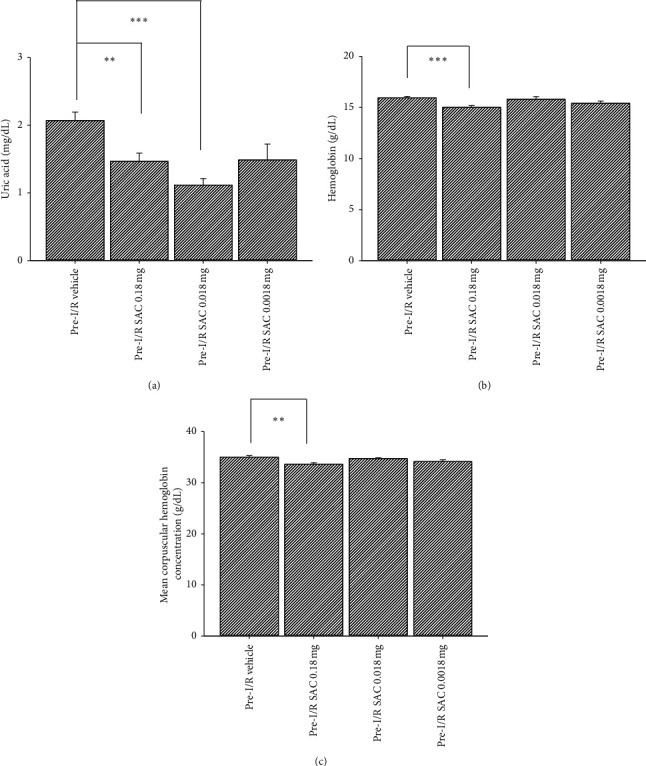
Blood parameter tests *after preischemia administration of vehicle versus various doses of SAC*. The bar charts show biochemical indicators of uric acid (a), Hb (b), and mean corpuscular Hb concentration (c) among the pre-I/R Vehicle, SAC 0.18 mg, SAC 0.018 mg, and SAC 0.0018 mg treatments. *∗∗*/*∗∗∗* represents significant difference (*P* < 0.01/0.001) from pre-I/R Vehicle. SAC, S-allyl L-cysteine.

Of note, the only significant side effects were the following parameters displayed in Figure 2, namely, uric acid, hemoglobin, and mean corpuscular hemoglobin concentration. For Figure 2(a), the pre-I/R vehicle group was not significantly different from the pre-I/R SAC 0.0018 mg group, but the pre-I/R SAC 0.18 mg and/or pre-I/R SAC 0.018 mg treatment resulted in significantly lower than normal levels of uric acid. This corresponds to auto analyzer values of 2.07 ± 0.13 vs. 1.47 ± 0.12 (*P*=0.005) vs. 1.12 ± 0.10 (*P* < 0.001) vs. 1.49 ± 0.23. As for Figure 2(b), the pre-I/R SAC 0.18 mg treatment caused slightly lower levels of hemoglobin, relative to that of pre-I/R vehicle group. These are associated with values of 15.95 ± 0.13 vs. 15.01 ± 0.18 (*P* < 0.001) vs. 15.81 ± 0.26 vs. 15.41 ± 0.22. Finally, the mean corpuscular hemoglobin concentration found in Figure 2(c) shows that pre-I/R SAC 0.18 mg treatment resulted in significantly smaller levels of hemoglobin, when compared with the pre-I/R vehicle group. The values obtained are as follows 35.02 ± 0.30 vs. 33.61 ± 0.31 (*P*=0.006) vs. 34.73 ± 0.18 vs. 34.15 ± 0.35.

### 3.5. The ERG b-Wave Ratio Changes after Pre- or Postischemia Administration of Medium/Low Dose of SAC

**Figure 3:**
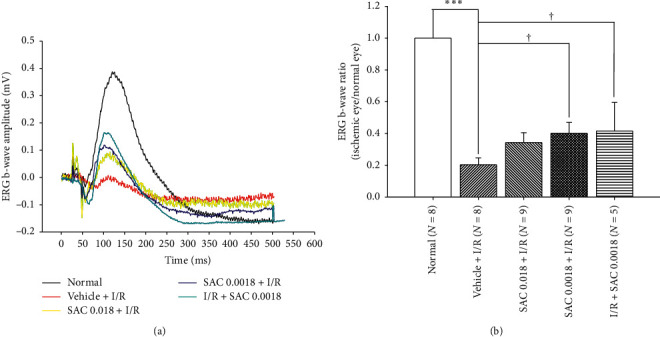
Pre- and postischemia administration of SAC protected the rat against retinal ischemia as seen by the ERG recordings. (a) The effect of SAC on the ERG b-wave amplitude of an eye subjected to ischemia/reperfusion (I/R). As compared with the normal group (normalized to 1), the b-wave amplitude decreased drastically in the Vehicle + I/R group that received I/R and pre-ischemia administration of vehicle. This ischemia-induced decrease was significantly alleviated by the preischemia administration of SAC (SAC 0.018/0.0018 mg + I/R) or postischemia administration of SAC (I/R + SAC 0.0018 mg). (b) After ischemia treatment, there was significant ERG b-wave ratio reduction, when the rat is provided with 4 weeks of preischemia daily oral ingestion of the same volume of vehicle (^*∗*^*P* < 0.001). As compared with Vehicle + I/R, the ischemia-induced ERG b-wave ratio reduction was significantly counteracted in the “SAC 0.0018 mg + I/R” or “I/R + SAC 0.0018 mg” group (^†^*P* < 0.05). The results are represented as mean ± S.E.M. (*n* = 5∼9). SAC, S-allyl L-cysteine; ERG, electroretinogram.

As shown in Figures 3(a) and 3(b), the HIOP-induced retinal ischemia significantly (*P* < 0.001) resulted in ERG b-wave ratio reduction of 0.20 ± 0.04 (*n* = 8), relative to that of normal (control) retina (ERG b-wave ratio = 1; *n* = 8). The ischemia-induced ERG b-wave reduction was either tended to be alleviated or significantly blunted by pre- or postischemia oral ingestion of SAC, namely, SAC 0.018 mg + I/R (0.34 ± 0.06, *n* = 9, *P*=0.11), SAC 0.0018 mg + I/R (0.40 ± 0.07, *n* = 9, *P* < 0.05), and I/R + SAC 0.0018 mg (0.42 ± 0.08, *n* = 5, *P* < 0.05).

### 3.6. Fluorogold Retrograde Labelling Alterations after Pre- or Postischemia Administration of Low Dose of SAC Are Given

**Figure 4:**
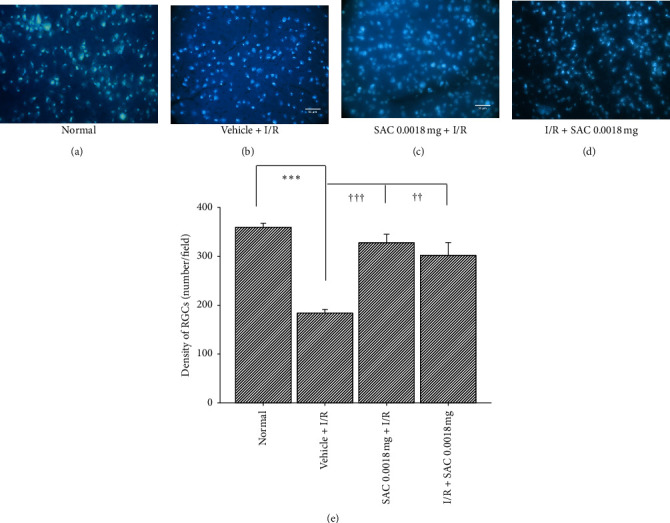
Fluorogold retrograde RGC immunolabeling. The microscopic images of RGC (as indicated by arrowheads) density of the following groups (*n* = 4), namely, normal (a), Vehicle + I/R (b), SAC 0.0018 mg + I/R (c), I/R + SAC 0.0018 mg, and (d) each bar represents the mean ± SEM of the density of RGCs (e). (^*∗∗∗*^*P* < 0.001) represents significant difference of Control vs. Vehicle + I/R, whereas †††/†† (*P* < 0.001/*P* < 0.01) indicates significant differences between Vehicle + I/R vs. SAC 0.0018 mg + I/R, or I/R + SAC 0.0018 mg. Scale bar = 50 *μ*m. SAC, S-allyl L-cysteine; RGC, retinal ganglion cell; I/R, ischemic plus reperfusion.

As demonstrated in Figure 4 (*n* = 4), the Vehicle + I/R treatment led to significant (*P* < 0.001) reductions in RGC numbers per field of 185.48 ± 7.50 (Figures 4(b) and 4(e)), relative to normal/control retina with a value of 361.23 ± 8.37 cells/field (Figures 4(a) and 4(e)). However, pre- or postadministration of 0.0018 mg of SAC was able to counteract the deleterious effects of I/R and led to a significant (*P* < 0.001/*P* < 0.01) elevated number of RGCs cells, namely, 329.52 ± 17.25 cells/field for SAC 0.0018 mg + I/R (Figures 4(c) and 4(e)) and 303.42 ± 26.52 cells/field for I/R + SAC 0.0018 mg (Figures 4(d) and 4(e)), relative to the Vehicle + I/R group.

### 3.7. Retinal Protein Levels of Wnt, HIF-1*α*, and VEGF after Pre- or Postischemia Administration of Low Dose of SAC

**Figure 5:**
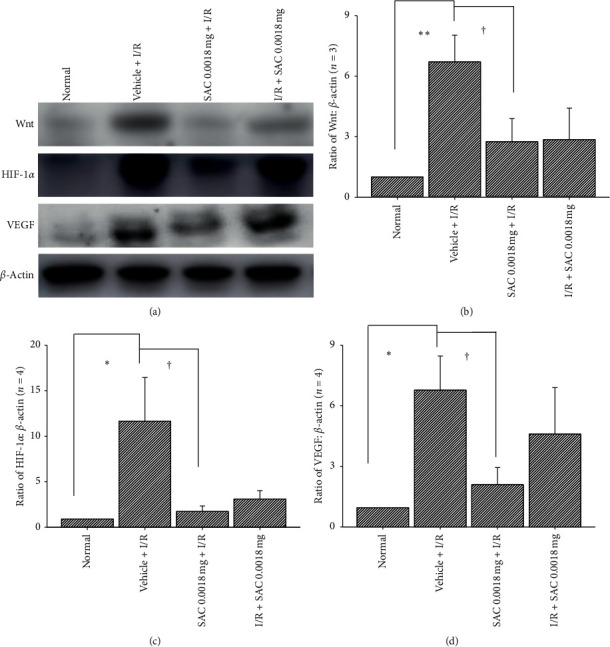
(a) Western blot analysis showing the expression levels of Wnt, HIF-1*α*, VEGF, and *β*-actin proteins. The blot images illustrated protein expression levels in normal retina as a control (column 1), whereas the 2^nd^ column illustrates ischemic retina preadministered with vehicle (Vehicle + I/R). Columns 3 and 4 showed a retina that received ischemia together with pre-/postadministration of 0.0018 mg SAC. Each bar in respective pictures represented the ratio of Wnt (b), HIF-1*α* (c), and VEGF to *β*-actin (d). *∗∗*/*∗* indicates a significant (*P* < 0.01/*P* < 0.05) difference between the normal retina and the ischemic retina preadministered with vehicle (Vehicle + I/R), whereas † indicated a significant (*P* < 0.05) difference between the “Vehicle + I/R” group and the ischemic retina preadministered with SAC (SAC 0.0018 mg + I/R). However, as compared with Vehicle + I/R, the ischemia-induced upregulation of Wnt/HIF-1*α*/VEGF had a trend to be, though not significantly, inhibited by the I/R + SAC 0.0018 mg treatment. The results are presented as mean ± S.E.M. (*n* = 4). SAC, S-allyl L-cysteine; HIF-1*α*, hypoxia inducible factor-1*α*; VEGF, vascular endothelium factor.

Compared with the normal control (normalized to 1; *n* = 3∼4; Figures 5(b)∼5(d)), there was a significant (*P* < 0.01/0.05) upregulation of target proteins, namely, Wnt3a (6.72 ± 1.32; *n* = 3; Figure 5(b)), HIF-1*α* (11.75 ± 4.80; *n* = 4; Figure 5(c)), and VEGF (6.82 ± 1.69; *n* = 4: Figure 5(d)) induced by retinal ischemia (Vehicle + I/R). In contrast, the pre- or postischemic oral gavage of SAC (SAC 0.0018 mg + I/R or I/R + SAC 0.0018 mg) significantly tends to be ameliorate the ischemia-induced significant overexpression of target proteins Wnt3a (**2.75** ± **1.15**, *P* < 0.05 or **2.86** ± 1.56, *P*=0.07; Figure 5(b)), HIF-1*α*(1.83 ± 0.59, *P* < 0.05 or 3.19 ± 0.92, *P*=0.06; Figure 5(c)), and VEGF (2.13 ± 0.85, *P* < 0.05 or 4.64 ± 2.30, *P*=0.2; Figure 5(d)). These results were also seen in the blot images (Figure 5(a)). Of note, the values above were presented as ratio of target protein to *β*-actin ± SEM.

## 4. Discussion

Retinal I/R is a pathological condition that leads to cellular damages in various ocular diseases, such as diabetic retinopathy, age-related macular degeneration, and glaucoma [13]. Ischemia makes the retina in a state that is hypersensitive to oxygen along with nutrients, triggering strong oxidative and inflammatory injury as the reperfusion is reinstated [20]. The neurons of retina are especially susceptible to these injures, which leads to features like retinal neurodegeneration [21]. In this study, retinal ischemic model induced by high IOP was used to provide insight into SAC's therapeutic properties and mechanism against I/R injury. Unlike other ligation techniques or models (e.g., compression of optic nerve or ophthalmic vessel by tying), I/R caused by elevated IOP gives a more targeted injury on the retina and causes minimal damage to the cornea [21]. Another consideration is that this method requires less surgical techniques and technological tools, which makes it more accessible than their alternatives, in terms of modelling retinal ischemic diseases [21].

The results show that I/R significantly affected the retina in a number of ways, via cellular viability (Figures 1 and 4), electrophysiology (Figure 3), and protein expression (Figure 5) evaluations, which is not inconsistent with that of the previous publications [13, 21]. MTT in vitro cellular viability has revealed significant RGC-5 cell loss following OGD insult (Figure 1), which also agrees with in vivo ischemia-associated decrease in number of fluorogold retrograde labelling RGCs (Figure 4). Note RGC cells is responsible for the transmission of all the visual information process by the retina to the brain and that the lack of RGC has a significant impact and is a common cause of irreversible blindness [22]. As for the electroretinography analysis, it documents I/R induced impairment of retinal neuronal function through lower b-wave amplitudes (reflected by Müller and bipolar cell activity), when compared with the non-I/R eye (Figure 3; [21]). Of note, ERG is a reflection of gross retinal physiologic function. Moreover, the death of retinal neurons (such as RGCs) reflects a pathological alteration. Lastly, the western blot analysis shows an upregulation of Wnt3a/HIF-1*α*/VEGF protein in the ischemic retina. In this case, it has been shown that HIF-1*α* and VEGF (angiogenic and vascular permeability protein) are target genes of Wnt pathway, which largely correlated with the development of ischemic-related ocular diseases [3].

Currently, it is said that lowering IOP reduces the risk of progressive RGC loss for most people, but a number of patient's vision still continue to deteriorate despite IOP treatment [23]. For instance, it is estimated that one out of eight patients will still become blind in one eye, which is attributed to glaucoma progression [24]. On the other hand, several treatments such as radiation, transpupillary thermotherapy, photodynamic therapy, and anti-VEGF antibodies are selected as treatments for ischemia-related ocular disorders, but these approaches are not completely effective when treating such vision threatening ischemic retinal disorders [14]. Disappointingly, poor visual outcomes have occurred among some patients after anti-VEGF or steroid treatment, even though ocular hemorrhage and macular edema have been successfully controlled. New treatments were involved in tackling different aspects of the problem, such as the upstream inhibitors of Wnt, which enhances the expression of HIF-1*α* and in return increases VEGF [13, 14]. These are required in order to effectively treat ischemic retinal disorders, when anti-VEGF or steroid treatment fails. The increased levels of Wnt (Figure 5(b)), HIF-1*α* (Figure 5(c)), and VEGF (Figure 5(d)) were detected during the present and previous studies. These are assumed to be linked in some way to retinal ischemia, wet AMD, and CRVO/BRVO. In such circumstances, based on the present results, SAC might provide an alternative way to deal with these ischemic-related vision-threatening retinal disorders and others, such as diabetic retinopathy.

Of clinical importance, our research showed that preischemia oral gavage administration of daily low dose of SAC (0.0018 mg) for 4 weeks was proven to be the safest and most effective, via observation of body weight changes (Table 1), ERG alterations (Tables 2 and 3), and blood biochemical parameters (see Results and Figure 2), as well as electrophysiological (ERG b-wave ratio; Figure 3) and/or histopathological results (density of RGC; Figure 4) that have supported for the protection of SAC against retinal ischemia. Specifically, both pre- and postischemic SAC significantly attenuated the ischemia-associated reduction of ERG b-wave and number of RGCs, thus demonstrating its neuroprotective properties [16]. However, the preischemic administration of SAC seems to be slightly more effective, when compared with the postischemic administration of SAC. This reasoning behind this trend will be discussed later in the next paragraph. In this case, it is well acknowledged that the formation of reactive oxygen species, namely, superoxide anion, hydrogen peroxide, and hydroxyl radical, is present (I/R) [20, 25], which can lead to pathological changes that occur at the cellular level (e.g., increased RGC cell mortality rate). Oxidative stress might currently play as another potential factor in ischemic-related ocular disorders, which is presently alleviated by SAC [7].

As for the novel anti-ischemic mechanisms of SAC, it might be explained by the significant downregulation of ischemia-induced Wnt/HIF-1*α*/VEGF overexpression (Figure 5), which is found in preischemic administration of the SAC 0.0018 mg + I/R group. On the other hand, there is no significant effect demonstrated in the postischemic administration of SAC; however, it had a trend of downregulating ischemia-associated increased Wnt/HIF-1*α*/VEGF protein levels. As mentioned before, activation of the Wnt signaling leads to overexpression of HIF-1*α* and VEGF levels in response to hypoxia [7–9, 11, 12, 26], which consequently results in the arrest of cell cycle [10], hindrance of cell proliferation, and increase in vascular permeability [27, 28]. This eventually leads to neuronal death [29], ocular hemorrhage, and macular edema [4]. Most of all, the present results have implied that preischemic fortified (low dose) SAC is able to electrophysiologically and histopathologically protect against retinal ischemia, via downregulating the ischemia-induced Wnt3a protein overexpression and consequently reducing levels of ischemia-associated HIF-1*α* and VEGF protein upregulation. Thus, SAC might be clinically useful as a preventative medication in defined retinal ischemia related disorders and possibly early developmental retinal vasculopathy.

Despite the therapeutic benefits it provides, it is important to note that concentrations higher than the proposed 0.0018 mg of SAC, such as 0.018 or 0.18 mg of SAC, were presently proved to cause side effects. The blood biochemical parameters were generally within the normal range for the low dose of SAC (see Results and Figure 3). However, the high (0.18 mg) dose of SAC significantly reduced the levels of uric acid (Figure 2(a)), hemoglobin (Figure 2(b)), and MCHC (Figure 2(c)), relative to non-SAC treatment. In addition, the medium (0.0018 mg) dose of SAC also resulted in significant lower than normal levels of uric acid but not for hemoglobin and MCHC levels. Indeed, consistent with the cellular viability test in Figure 1(b), any concentration above 1 mM (in vitro) or 0.0018 mg/day (an in vivo equivalent) of SAC, it starts to lose its protective effect and no longer shows a dosage related attenuation against ischemic injury. In other words, its toxic effect [17] becomes amplified at higher concentrations, and higher doses of SAC might paradoxically alter its biochemical properties [30].

Overall, the present results help us in the understanding of the mechanisms involved when retinas are subjected to I/R. In addition, these results have supported that preischemia administration of low-dose SAC for 4 weeks is not only safe but also alleviates defined harmful ischemia-associated findings [7–9, 11, 12, 26], namely, electrophysiologic dysfunction, RGC death, and upregulated defined proteins (e.g., vessel development related Wnt) [4–6, 31–33]. This study has provided insights into the therapeutic effect and safety of SAC consumption as a new potential treatment method for prevention of ischemia-related ocular diseases.

## 5. Conclusion

Preischemia administration of fortified SAC (low dose: 0.0018 mg) is proven to be experimentally safe as shown by the animal safety experiments and electrophysiological/histopathological data, in terms of the ability of SAC to alleviate retinal ischemia injury. Of medical importance and novelty to this paper, the ischemia-induced elevation of upstream protein Wnt3a and its associated downstream target HIF-1*α* and VEGF protein levels were significantly downregulated by 4 weeks of preischemic administration of fortified SAC but not by vehicle. Therefore, SAC might safely provide an alternative method of preventing vision-threatening ischemia/Wnt-related ocular disorders (e.g., familial exudative vitreoretinopathy), relative to conventional treatment methods such as anti-VEGF.

## Figures and Tables

**Table 1:** Body weight (g) changes after oral gavage for 4 weeks of preischemia daily doses of Vehicle or SAC treatment.

Time/group	Vehicle	SAC 0.18 mg	SAC 0.018 mg	SAC 0.0018 mg
Baseline	220.2 ± 18.1	217.0 ± 18.2	221.8 ± 16.9	224.0 ± 18.1
Week 1	258.2 ± 16.0	257.3 ± 14.5	262.2 ± 15.8	269.5 ± 18.2
Week 2	282.2 ± 13.8	280.0 ± 8.3	304.5 ± 14.7	292.0 ± 12.1
Week 3	324.0 ± 12.3	310.7 ± 6.7	325.7 ± 9.1	328.2 ± 8.2
Week 4	321.0 ± 9.5	322.3 ± 8.7	323.3 ± 12.5	330.7 ± 17.7

Body weights (BWs) were measured at the baseline (0 week) or 1/2/3/4 weeks after oral gavage of preischemia administration of daily doses of vehicle or SAC (0.18, 0.018 or 0.0018 mg) treatment. The results are recorded without following ischemia and represented as mean ± S.E.M. (*n* = 6∼11). SAC, S-allyl L-cysteine.

**Table 2:** The effect on electroretinogram a-wave amplitudes (mV) changes after oral gavage for 4 weeks of preischemia daily doses of SAC or Vehicle treatment.

Time/group	Vehicle	SAC 0.18 mg	SAC 0.018 mg	SAC 0.0018 mg
Baseline	0.08 ± 0.02	0.06 ± 0.02	0.09 ± 0.02	0.07 ± 0.02
Week 1	0.01 ± 0.03	0.09 ± 0.02	0.07 ± 0.02	0.05 ± 0.01
Week 2	0.09 ± 0.03	0.08 ± 0.02	0.10 ± 0.02	0.06 ± 0.02
Week 3	0.07 ± 0.02	0.07 ± 0.02	0.06 ± 0.02	0.06 ± 0.02
Week 4	0.09 ± 0.03	0.07 ± 0.02	0.09 ± 0.02	0.06 ± 0.01

**Table 3:** The effect on electroretinogram b-wave amplitudes (mV) changes after oral gavage for 4 weeks of preischemia daily doses of SAC or Vehicle treatment.

Time/group	Vehicle	SAC 0.18 mg	SAC 0.018 mg	SAC 0.0018 mg
Baseline	0.43 ± 0.03	0.41 ± 0.05	0.48 ± 0.06	0.41 ± 0.07
Week 1	0.49 ± 0.05	0.44 ± 0.05	0.44 ± 0.06	0.45 ± 0.06
Week 2	0.43 ± 0.07	0.37 ± 0.06	0.46 ± 0.10	0.47 ± 0.08
Week 3	0.41 ± 0.07	0.35 ± 0.04	0.44 ± 0.06	0.40 ± 0.01
Week 4	0.35 ± 0.04	0.38 ± 0.05	0.44 ± 0.03	0.41 ± 0.04

The ERG amplitudes of a- and b-waves were recorded at the baseline (0 week) or 1/2/3/4 weeks after oral gavage of daily doses of vehicle or SAC (0.18, 0.018, or 0.0018 mg). The results are recorded without following ischemia and represented as mean ± S.E.M. (*n* = 6∼11). ERG, electroretinogram; SAC, S-allyl L-cysteine.

## Data Availability

The data used to support the findings of this study are available from the corresponding author upon request.
